# The neural impact of editing on viewer narrative cognition in virtual reality films: eye-tracking insights into neural mechanisms

**DOI:** 10.3389/fpsyg.2025.1584250

**Published:** 2025-08-29

**Authors:** Qiaoling Zou, Wanyu Zheng, Zishun Su, Li Zhang, Ziqing Zhuo, Dongning Li

**Affiliations:** ^1^School of Design, Jiangnan University, Wuxi, China; ^2^School of Digital Technology & Innovation Design, Jiangnan University, Wuxi, China; ^3^School of Art Design, Shanghai Business School, Shanghai, China; ^4^School of Art, Wuxi Taihu University, Wuxi, China

**Keywords:** virtual reality, eye-tracking technology, film editing, narrative cognition, visual behavior, neurocinematics

## Abstract

**Introduction:**

The development of virtual reality (VR) films requires novel editing strategies to optimize narrative cognition in immersive environments. While traditional film editing guides attention through controlled sequences of shots, the interactive nature of VR disrupts linear storytelling, challenging creators to balance emotional experience and spatial coherence. By combining eye-tracking technology with neuroscientific findings, this study aims to investigate how different editing techniques in virtual reality (VR) films affect viewers’ narrative cognition, focusing on visual attention, emotional experience and cognitive load, and to optimize VR film editing strategies through a neurocognitive lens.

**Methods:**

A controlled experiment with 42 participants was conducted using three versions of a VR movie: an unedited movie, a hard cut edited movie, and a dissolve-transition edited movie. Eye-tracking metrics were recorded using the HTC Vive Pro Eye headset, and emotional experiences were assessed using post-viewing questionnaires. Data were analyzed using SPSS and visualized using heat maps and trajectory maps.

**Results:**

The unedited movie (F1) elicited the highest visual attention (TDF: *M* = 18,953.83 vs. F2/F3, *p* < 0.001) and emotional immersion, with 75% of viewers rating it as “highly immersive.” It also showed sustained activation in areas related to emotional engagement. Edited movies, both hard cuts (F2) and dissolve-transitions (F3), reduced cognitive load (TSD: *M* = 16,632.83 for F1 vs. 15,953.18 for F3, *p* < 0.01) but resulted in fragmented attention. Dissolve-transitions (F3) decreased viewer enjoyment (APD: *M* = 0.397 vs. F1, *p* < 0.001). One-way ANOVA analysis revealed that seamless editing enhanced emotional coherence, while abrupt cuts disrupted spatial and temporal integration, leading to reduced emotional engagement.

**Discussion:**

Unedited VR films promote emotional coherence driven by the amygdala and maintain attention stability mediated by the prefrontal cortex, which enhances immersive narrative cognition. In contrast, editing techniques prioritize cognitive efficiency at the expense of emotional experience. To maintain immersion, filmmakers should focus on seamless transitions, while strategically using edits to direct attention in the complex 360° environment of VR. These findings contribute to neurocinematic theory by connecting the neural dynamics induced by editing with behavioral outcomes, offering practical insights for VR content creation.

## 1 Introduction

The rapid evolution of virtual reality (VR) technology has led to radical shifts in the domain of film production, especially in terms of narrative structure and viewer perception. By breaking the traditional two-dimensional narrative structure, VR establishes an interactive and immersive environment for the viewer. In this environment, the viewer is granted autonomy to select their preferred viewing perspectives (Serrano et al., 2017b). As director Doug Liman stated, “… we had to rethink the way we were telling stories, because when you just take a traditionally scripted scene out of any TV script or movie script and shoot it in VR, it’s going to be less compelling than what was shot in 2D” (Robertson, 2016). This transformation requires filmmakers to re-examine how to influence the viewer’s cognitive and emotional experience through audiovisuals during the production process, especially in an immersive environment where the viewer can actively select perspectives. Ultimately, the immersive nature of VR has profound implications for neural processing, as it engages multiple sensory modalities and cognitive systems simultaneously, potentially altering how the brain encodes and retrieves narrative information (Slater and Sanchez-Vives, 2016b).

Narrative cognition in film is crucial for the viewer. It is not only the foundation for understanding the storyline and character relationships but also the core driving force behind emotional resonance and immersive experience. Through narrative cognition, viewers are able to integrate scattered audiovisual information into a coherent mental representation, constructing the spatiotemporal logic and causal chains of the story world. However the viewer’s narrative cognition of a film is a complex process (Cutting, 2016) involving cognitive processing of aspects such as time, space, causality, character emotions, and multiple narratives (Bortolussi and Dixon, 2003; Kovács and Papp-Zipernovszky, 2019; Thompson, 1999). “VR is different from other forms of human–computer interface since the human participates in the virtual world rather than uses it, there is a clear role also for immersive movies, where the participant plays a role within the story” (Slater and Sanchez-Vives, 2016a). This allows them to engage with the story from various angles and perspectives. This interactivity and sense of space overrides the linear narrative of traditional film, allowing a deeper experience (Aylett and Louchart, 2003). In order to adapt to this new mode of viewing experience, the editing techniques of VR films need to change fundamentally. The director of a traditional film can control the pace of the narrative by editing, which in turn promotes the narrative of the film (Skorin-Kapov, 2019). But in a VR film, viewers can change their perspective and position as they want, so their field of view is not controlled by the director. Consequently, the linear narrative structure no longer applies to VR films (Dooley and Munt, 2024). Creators need to rethink how to change scenes and share information in VR environments.

Editing is regarded as a pivotal element in the film-making process, playing a crucial role in the construction of narrative and the shaping of film style. “The intention of most film editing is to create the impression of continuity by editing together discontinuous viewpoints” (Smith, 2012). Shots, being “the smallest film units to which viewers are asked to direct their attention” (Cutting et al., 2010), are meticulously arranged through skillful editing to imbue a film with coherent narrative rhythm, thereby influencing the story’s trajectory and significantly affecting the viewer’s comprehension and emotional response (Gabert-Quillen et al., 2015). Editing not only modifies the sequence of events and disrupts the linear narrative structure, but also establishes intricate causal relationships, enabling the viewer to gather more information through indirect narration. The regulation of shot length and switching frequency is instrumental in shaping the film’s pace and directing the viewer’s attention (Smith, 2006). Through the manipulation of emotional tension and the pacing of events, filmmakers can evoke intense emotional responses in the viewer. Accelerating the pace of events creates heightened emotional intensity, characterized by urgency and anticipation, while slowing down the narrative generates moments of calm and composure. The dynamics of attention shift in response to emotional and narrative stimuli (Bezdek and Gerrig, 2017). Precise manipulation of pacing guides the viewer through the story’s progression, evoking a range of emotional states, and profoundly influencing their understanding of the film’s narrative content. More importantly, these editing strategies have been shown to elicit specific neural responses: increased activity in the prefrontal cortex during attention shifts and significant activation of the amygdala during emotional climaxes (Stangl et al., 2023).

However, most research focuses on how to create immersive experiences (Ross and Munt, 2018) and interactive mechanisms (Tong et al., 2021) in VR environments currently. In contrast, there are few studies on how to optimize the narrative effect of VR films through editing techniques. This suggests that the current academic understanding of VR film editing is limited, especially in terms of how to adapt the editing strategy to the cognitive characteristics of the viewer’s visual behavior. Many unknown areas still require further investigation. For example, how does the neural activity in brain regions related to attention control and emotional regulate during VR film editing? How are these neural responses associated with the viewer’s narrative understanding and emotional engagement? This study aims to investigate the neural effects of editing in VR films through eye-tracking technology, with a particular focus on how it influences viewers’ narrative cognition. Eye movement can not only precisely reflect the viewer’s attention focus and gaze distribution, which are closely related to the neural activity of the visual cortex and frontal cortex (Henderson, 2003), but also can provide key evidence for the analysis of the neural processing mechanism of narrative information. The experimental data can be analyzed to gain insight into the impact of editing on viewer gaze distribution, attention focus, and narrative comprehension. Finally, this research provides a scientific basis for optimizing VR film editing, enhancing the viewer’s narrative understanding, and providing potential applications in media production by considering cognitive processes and viewer engagement.

Based on the background and the basic objectives of the study, this research proposes the following questions:

*Q1*. To what extent does editing affect viewers’ narrative comprehension in a VR environment?

*Q2*. What kind of editing in VR films can help viewers improve their cognition and memory of the narrative?

*Q3*. Do viewers show different visual behavior in VR films with different editing styles? How do eye-tracking data reveal these differences?

*Q4*. How to adjust the VR film editing strategy according to the viewer’s visual behavior and cognitive characteristics to optimize the narrative effect and viewer experience of VR films?

## 2 Literature review

This study uses a narrative literature review to systematically organize and synthesize how editing techniques in VR films influence viewer narrative cognition through neural mechanisms, under the interdisciplinary background of cognitive neuroscience, VR production technology, and eye-tracking. To improve the structure of the review, we adopted the SALSA framework proposed by Booth et al. (2021). This framework can make narrative reviews more strict by guiding the literature search, critical appraisal, synthesis of findings, and analytical interpretation. Within this collective framework, we first created a general idea of the research topic through a wide literature search that is not limited to any single discipline. We then conducted an in-depth synthesis of relevant theoretical models and empirical studies along three dimensions: narrative cognitive neural mechanisms, the evolution of VR editing technology, and eye-tracking and cognitive processing. The three dimensions systematically support the research chain from VR film editing through neural mechanisms to viewer narrative cognition. Finally, by interdisciplinary comparison and analysis, we uncovered the intrinsic connections and potential gaps between different research perspectives.

### 2.1 Narrative cognition theory and its neuroscientific perspective in VR film

Cognitive narratology emerged from the cognitive revolution of the mid-20th century, when scientists began to systematically study human thought processes. The early cognitive scientist (Neisser, 2014) focused on the mechanisms of memory and information processing, showing the internal processes about how the senses, after receiving stimulus inputs, perform mental operations such as transformations, simplifications and refinements in order to acquire, store and use knowledge. With more study of language and narrative, narrative has come to be regarded as a key cognitive structure that plays an important role in language development, education, and psychological processes (Graesser, 1993). At the end of the 20th century, narrative theory was further integrated with cognitive science and became a fundamental way of human to think about problems, understand the world and communicate with each other (Turner, 1996). It is not just a literary or linguistic form, but a cognitive tool that helps people understand complex concepts such as time, space, cause and effect, and role motivation (Herman, 2007). Bruner (1991) thought that narrative cognition, as a way of interpreting the world, can help individuals construct themselves and understand others in an ever-changing environment (Herman, 2004), on the other hand, saw narrative cognition as a framework for organizing experience and knowledge. He claimed that narrative can help people understand and explain the complex world through causality, chronology and character interactions.

In the context of VR film, narrative cognition becomes more complex as it operates in an interactive, immersive environment. Viewer understanding and experience is seen as the basis of narrative perception. Prince (2003) introduced the concept of the ‘narratee’, which suggests that narratives are not simply conveyed from the narrator to the viewer, but are constructed within a communicative framework that is predetermined by the viewer. Viewers use narrative as a cognitive tool to construct chains of cause and effect in a complex reality, and to understand and anticipate the development of future events through logical reasoning. Rimmon-Kenan (2006) emphasized the cognitive involvement of the viewer in the narrative process, that is, the viewer is not just a passive recipient of information, but also an active participant in the process of construction of the story through reasoning, imagination and anticipation. Margolin (1990) argued that the characters in a narrative story are constructed by the viewer through clues taken from the text. Lanser (1981) proposed a focus on the study of readers’ construction of authorial representations and motivations in narrative research from a discourse theory perspective. Audience-centered narrative cognition theory research provides new perspectives on how VR film the viewer construct and understand stories in virtual environments.

Visual attention, emotional experience and cognitive load are three important metrics to quantify the narrative cognition of the viewer (Skaramagkas et al., 2021). Firstly, attention is a basic cognitive function and the ability to focus on most useful information in a complex environment (Wolfe, 1999). And the term ‘visual attention’ refers to a set of cognitive operations that mediate the selection of relevant and the filtering out of irrelevant information from cluttered visual scenes (Seikavandi and Barret, 2023). Therefore, visual attention is fundamental to the viewer’s understanding of a VR film story. Secondly, there is an interactive relationship between cognition and emotion (Liu et al., 2009). “Emotions can constitute reasons for our beliefs and judgments, or can provide information about our evaluative situation” (Brady, 2013). So emotional experience plays a key role in viewer’s understanding of the narrative. Finally, cognitive load is generally seen as the product of the factors that influence the efficiency of a person’s workload in a task (Wickens, 2008). It can impact the perception of VR films narrative significantly (Sweller, 1994). As a result, the correct control of cognitive load in VR films is also important to increase viewer understanding and experience.

From a neuroscientific perspective, these cognitive processes are closely linked to neural mechanisms. Visual attention involves brain regions such as the visual cortex and prefrontal cortex, which are responsible for processing and directing attention (Corbetta and Shulman, 2002). Emotional engagement activates regions such as the amygdala, which regulates emotional responses, and the prefrontal cortex, which is involved in decision-making and emotional regulation (Bechara et al., 2000). In addition, cognitive load affects working memory and cognitive control networks, including the dorsolateral prefrontal cortex, which is responsible for managing mental resources during tasks (Duncan, 2010). In VR films, the interaction between these cognitive functions and neural responses provides us with profound insights into how editing and immersive environments affect viewer comprehension, emotional engagement and memory retention. Beijing Normal University has made significant contributions in this area. Wang’s team carried out studies combining behavioral and neuroimaging experiments to explore how film elements like color and editing influence viewers’ emotional perceptions together. Their interdisciplinary method connected cognitive neuroscience and film studies, increasing our understanding of narrative cognition in VR environments (Cao et al., 2024). Mastnak (2024) has published work titled “Neurocinematic Therapy-An Interdisciplinary Perspective.” He introduced “neurocinematic therapy” as an applied branch of neurocinematics. This approach emphasizes viewers’ neuropsychological responses to films, exploring how film experiences can influence emotional regulation and mental health. He discussed the interaction between psycho-affective phenomena and central nervous system functions, highlighting how films can be used as a treatment in medical settings.

### 2.2 The evolution of editing technology in VR film

Editing techniques have changed since the birth of film. In the 1930s, Eisenstein et al. (1989) proposed the theory of ‘montage’, arguing that film could create new meaning and emotion by combining images. After that, Cut (1977) used the jump-cut technique to reshape the viewer’s perception of time and space in a linear narrative. This attempt successfully increased the sense of reality and viewer participation in the film. Since the 21st century, the advent of digital film has given creators a higher level of control. Meanwhile, film editing has become not only a narrative method but also an important way to express emotion and present thought (Lefebvre and Furstenau, 2002) for the creator. It has undergone a real transformation in the VR era. It is no longer just a process of connecting different shots, but has become a tool for shaping the viewer’s experience.

Traditional films use editing techniques to build narrative tension, create character relationships, and show temporal and spatial changes. It guides the viewer’s perception of the story by creating a coherent narrative flow (Eisenstein, 2014). In VR film, however, the editing technique completes the shift from linear to interactive narrative. It allows viewers to actively construct the narrative experience through their choices, rather than passively accept the meaning given by the director (Fencott, 2003). Early pioneers like Brillhart (2016) identified core challenges in VR editing, arguing that abrupt cuts like the “floating head cut” disrupt spatial presence and immersion. She conceptualized VR editing as “not so much a putting together as it is a discovery of a path,” critiquing traditional continuity methods (e.g., eyeline match, match on action cuts) for risking spatial discontinuity. This concern was empirically substantiated by MacQuarrie and Steed (2018), who demonstrated that transition types significantly alter subjective spatial motion perception. Extending this work, MacQuarrie and Steed (2020) highlighted a critical physiological constraint: in 360° environments, vergence mismatch, where virtual characters’ eyes fail to converge at the viewer’s position, can cause gaze-direction confusion and even misperceptions of strabismus, thereby undermining realism and narrative engagement. To address the foundational failure of traditional continuity editing in VR, Serrano et al. (2017b) leveraged cognitive event segmentation theory, systematically validating continuity perception mechanisms across edit boundaries. Their findings revealed that spatial misalignment promotes exploratory viewing behavior and proposed novel attention metrics, providing empirical foundations for VR editing paradigms aligned with audience cognition.

The VR film editing is difficult to maintain spatial orientation and narrative stability. And it’s more complex to guide the viewer’s attention in virtual reality, because they can explore the environment and change their viewpoint as they want freely (Speicher et al., 2019). This freedom significantly increases the audience’s cognitive load, such as spatial disorientation, leading to impeded narrative understanding and a decline in emotional engagement (Cao et al., 2019). Therefore, some researchers emphasized that the VR film editing must solve the problem that how to orient the space through visual cues and environmental design (Neo et al., 2021). Critical to this effort are rules like the 30-degree principle (avoiding jarring perspective shifts less than 30) and 180-degree axis maintenance (Zhang C. et al., 2024), which stabilize spatial cognition by aligning cuts with viewers’ embodied perception.

However, the effectiveness of these cues depends not only on their ability to provide a coherent narrative in a virtual environment, but also on their capability to create a fully immersive experience for the viewers (Slater and Wilbur, 1997). A full immersion experience can draw a strong emotional response from the viewer (Hartmann and Fox, 2021), and emotional engagement is a key part of narrative understanding. Therefore, to combine VR editing techniques with the immersion of virtual reality, creators need new editing models for the specific features of virtual reality (Brown and Lowe, 2007). Recent frameworks advocate intra-shot editing, such as dynamic gaze guidance, diegetic cues, virtual frame cutting (using in-scene elements as focal guides), or spatial montage (simultaneous layering of perspectives) to replace inter-shot cuts and minimize spatial disruption (Matkin, 2019). Parallelly, use the CVR long take to continuous shots preserving spatiotemporal unity emerges as a strategy to maximize immersion, aligning with findings on unedited VR’s emotional potency. They have to re-evaluate traditional editing techniques and create new ones to establish narrative structure and viewer engagement (Dooley, 2017). Thus, future research should focus on the development and refinement of VR editing techniques. Such methods can improve the viewer’s understanding of the narrative while providing a more immersive experience. Zhang and Weber (2023) suggested to explore new forms of editing applied to interactive narrative. They thought that the immersive capabilities of virtual reality can create more engaging and coherent narrative content.

### 2.3 Eye-tracking technology and cognitive processing

In recent years, eye-tracking technology has been widely used in film theory research. It can accurately record the gaze point, gaze duration, pupil size, and other data of human eyes, thus helping researchers to understand the viewer’s attention allocation, emotional response, and cognitive processes when watching VR films (Duchowski, 2002; Velichkovsky et al., 1996). In traditional film studies, eye-tracking techniques can be used to evaluate a film’s audiovisual effects (Swenberg and Eriksson, 2018; Romero-Fresco, 2020), narrative structure (Markhabayeva and Tseng, 2024) and viewer emotional response (Swenberg et al., 2022). However, using eye-tracking technology in VR environments is not easy, mainly because the high degree of freedom and immersive scenarios of VR films make it difficult to collect data. At the same time, VR environments can lead to eye fatigue and dizziness, resulting in blurred vision, which in turn affects the perception of size and distance of objects in the virtual environment (Eggleston et al., 1996). Early VR eye-tracking studies mainly used desktop VR systems, and with the development of head-mounted display device (HMD), the combination of HMD and eye tracker has greatly improved the accuracy of the data and the naturalness of the experiments (Lin et al., 2003). HMD devices with integrated eye-tracking technology, such as the HTC Vive Pro Eye and Oculus Rift S, are now widely used in VR film research. These devices can record the viewer’s point of view and line of sight when watching a 360-degree VR film, providing a more complete perspective for analyzing the viewer’s behavior in a virtual scene (Sipatchin et al., 2021).

Immersion is a key area of research in VR films, as it directly influences viewers’ experience, emotional engagement, and ultimately their narrative comprehension. Eye-tracking can reveal the relationship between viewers’ attention distribution and their level of immersion (Hoffman and Subramaniam, 1995). Torralba et al. (2006) argued that eye-tracking is an effective tool for understanding gaze distribution during natural viewing. Clay et al. (2019) proposed that eye-tracking can provide valuable insights into user experience in VR environments by detecting viewers’ attention levels and information processing channels. Further research has demonstrated that immersive experiences in virtual reality are closely linked to viewers’ visual attention patterns. Users who are more engaged tend to focus on key narrative elements for longer duration, indicating deeper attention and emotional involvement (Ding et al., 2018). Waltemate et al. (2018) further confirmed that users show less visual distraction and more focused attention in highly immersive virtual environments. Eye-tracking technology can be used not only to understand viewers’ visual behavior in virtual reality but also to reveal their attention patterns in complex narrative structures. Bailenson et al. (2004) found that when the viewer join various forms of social interaction in virtual environments, their visual behaviors and level of immersion are significantly influenced by narrative styles. These studies provide a solid empirical foundation for understanding viewer experiences in VR films.

Based on previous literature and the formulated research questions, the following hypotheses are proposed:

*H1*: The application of editing techniques can disrupt viewers’ attention focus in VR films.

*H2*: The implementation of editing techniques can diminish the emotional engagement of the viewer in VR films.

*H3*: The use of appropriate editing techniques can reduce the cognitive load experienced by the viewer while viewing VR films under specific conditions.

## 3 Materials and methods

### 3.1 Experimental design

**Figure 1:**
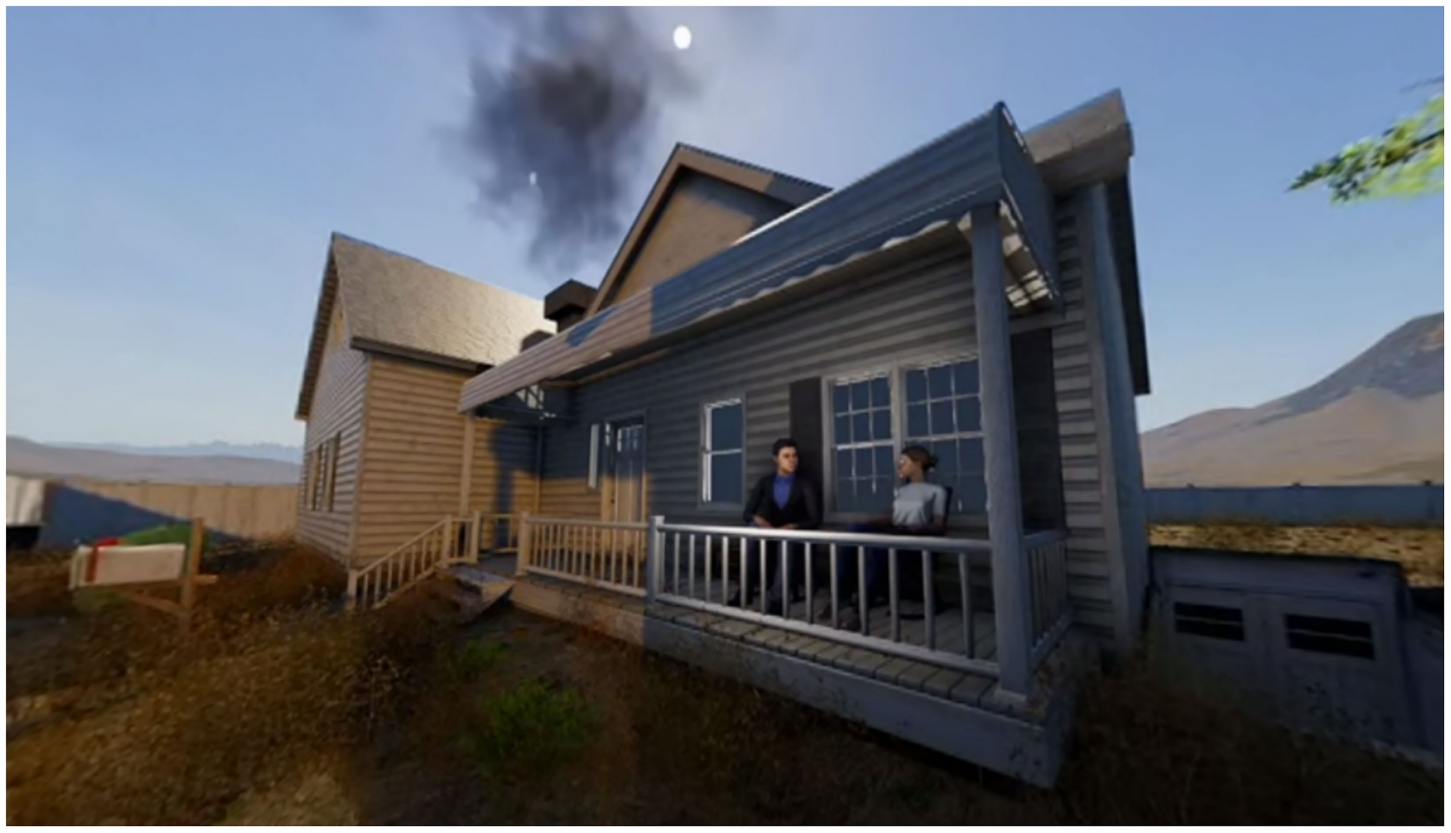
Virtual reality (VR) movie *Tornado and storm survival*.

Eye-tracking technique was used in this study to test the research hypotheses. Firstly, we picked a VR movie: *Tornado and storm survival* on YouTube (Figure 1). This is a science popularization VR movie created by Bright Side VR, a team specializing in 360° content production. It is one of the team’s core productions. This film was chosen as the experimental material for this study due to its ability to provide highly immersive disaster experiences through immersion and presence. Using realistic CGI effects and 360° panorama technology, the film enables viewers to experience the destructive power of a tornado in a safe environment, which is a unique advantage over traditional 2D media. Produced to a high standard, the film is a classic example of the immersive appeal of VR technology, with carefully designed scenes that maintain spatial continuity. The strong sensory stimulation and narrative approach of this educational film visually convey the plot and meaning, making it easy for the audience to understand. At the same time, the film is easily accessible and meets the requirements for experimental material. It guides the storytelling through a strong and easy narrative structure, clear viewer behavioral direction, and well-defined narration. The movie was shot using a long-take technique with no editing effects. Then, three versions of the film, labeled F1, F2, and F3, were generated as experimental materials. F1 was the original film without any editing applied. In this version, viewers needed to actively search for the main narrative areas (the key storytelling regions) within the 360° field of view of the VR movie.

F2 utilized hard cuts for the editing process. This technique was selected as the most fundamental and disruptive form of transition in film editing, involving an instantaneous shift from one shot to the next with no visual overlap. Within the specific context of VR, hard cuts have been consistently identified as particularly problematic for spatial continuity and viewer immersion (Zhang C. et al., 2024; Serrano et al., 2017b; Brillhart, 2016). Their abrupt nature can cause significant spatial disorientation and attentional disruption in 360° environments, making them a critical case study for understanding the potential negative neural and cognitive impacts of conventional editing techniques in VR.

F3 employed dissolve transitions, characterized by a gradual blending of one shot into the next over a period of time. Dissolves are a common technique in traditional cinema often used to signify a softer transition, a passage of time, or a connection between scenes. While potentially less jarring spatially than hard cuts in 2D film, their application in VR introduces unique considerations. Recent research suggests that dissolves, due to their temporal overlap and potential for visual blending across disparate spatial locations, can still disrupt spatial coherence and may even prolong the period of attentional reorientation compared to a seamless flow, potentially increasing cognitive load in a different way (Serrano et al., 2017b; MacQuarrie and Steed, 2020). Their inclusion allows us to compare a “smoother” traditional transition against both the unedited movie (F1) and the maximally disruptive hard cut (F2).

According to Murch (2001), one of the most important functions of editing is to guide the viewers’ attention and direct their visual and emotional focus toward the most important parts of the story. The core manipulation defining F2 and F3 relative to F1 was that editing was used to explicitly direct the viewer’s gaze to the main narrative areas at specific points, eliminating the need for active search within the 360° space. Crucially, the type of transition used (hard cut vs. dissolve) allowed us to investigate how the manner of this explicit attentional guidance impacts the hypothesized outcomes (H1: Attractiveness, H2: Emotional Engagement, H3: Cognitive Load), particularly in light of the known challenges these transitions pose to VR spatial cognition and continuity. This version of the movie was edited using 5 cut points, designed according to the changing positions of the main narrative areas. It also used hard cuts without any special effects. These points are, respectively, located at 00:11, 00:16, 00:31, 00:38 and 1:16. F3 was edited using a dissolve effect, and conforms to the 30° and 180° rules, which was the same as F2’s cut points, only the clip transition method was different.

### 3.2 Participants

The research group randomly recruited 42 volunteers between the ages of 18 and 36 to participate in this study. A total of 25 were male and 17 were female in the experiment. All the volunteers were current students at Jiangnan University. They had normal cognitive and motor abilities, no history of mental illness or substance abuse, and were not majoring in film or any related discipline. The research required participants to have normal vision or corrected-to-normal vision without eye disease such as color blindness or color weakness. All participants signed an informed consent form to protect the rights of volunteers. All data was collected in a way that protects the privacy of the individual and ensured that no identifying information about the participant was disclosed. If the article includes images of the participants, written consent must be obtained from the participants. The demographic characteristics of participants are shown in Table 1.

**Table 1:** Demographic characteristics of participants.

Category	Type	Sample count	Percentage
Gender	Male	25	59.5%
	Female	24	40.5%
Age range	18 ~ 24 years old	21	50.0%
	24 ~ 30 years old	15	35.7%
	30 ~ 36 years old	6	14.3%
Familiarity with VR technology	Never did	3	7.1%
	A little	32	76.2%
	Very well	7	16.7%
Familiarity with VR films	Never did	13	31.0%
	Occasionally	26	61.9%
	Often	3	7.1%

It shows that most participants had experience watching VR films and could watch and interact with them on their own. To make sure that everyone understood and could interact with the experimental materials, those with no prior VR film viewing experience received training before the formal experiment. We showed them five VR films of different types.

### 3.3 Experimental procedures

The experimental procedures were conducted individually in an enclosed well-conditioned laboratory space. Before the experiment began, each participant was required to sign an experimental consent form and fill out a questionnaire with basic information about their gender, age, and VR proficiency. During this process, participants were observed and engaged in brief conversations to ensure that they were in a normal emotional and psychological state for the test, and that they did not have symptoms such as depression, irritability, or excessive excitement. Next, they were led to get familiar with the site and wear the VR HMD. After calibrating the eye-tracker in the HMD, the experiment started according to the following steps:

1. Participants were informed of the experimental procedure and told to terminate the experiment immediately if they felt any discomfort such as dizziness. In this step, participants were informed of the purpose of our experiment and were instructed to focus specifically on the narrative aspects of the VR movie.
2. Participants received visual calibration to ensure the accuracy of the eye-tracking data.
3. Participants watched VR films F1, F2, and F3 in random sequence. The following six combinations were played in random order: F1F2F3, F1F3F2, F2F3F1, F2F1F3, F3F1F2, F3F2F1.
4. After viewing, participants filled out a questionnaire to rank and rate the three movies in terms of visual fluency, content clarity, visual comfort, and immersion.

This study was conducted in accordance with the Declaration of Helsinki and approved by the Institutional Review Board of Jiangnan University. At the end of the experiment, participants received thanks and a gift in return. Two facilitators were present during the experiment to ensure proper conduct of the experiment. They also provide information and services as needed, including helping participants to wear the VR equipment correctly.

### 3.4 Experimental equipment

**Figure 2:**
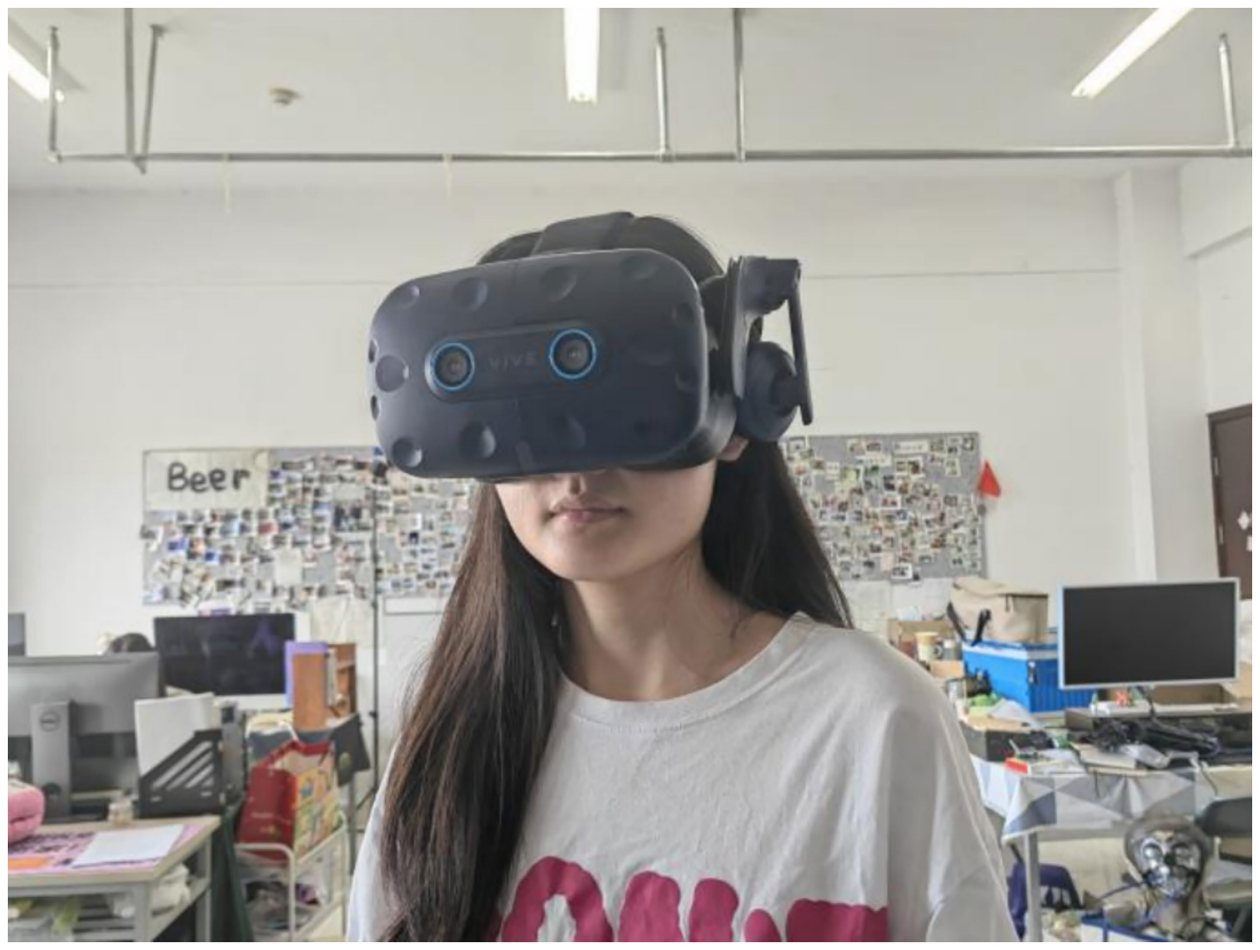
Participants were using the HTC Vive Pro Eye.

This study employed Tobii Pro Lab and HTC Vive Pro Eye VR (Figure 2). They were used to collect and analyze eye movement data in order to explore the effects of VR image editing on viewers’ narrative perceptions. Tobii Pro Lab is an advanced eye-tracking data analysis software for research and business. It supports high-precision real-time data acquisition and complex experimental design. This software handles VR movie stimuli efficiently and is compatible with the HTC Vive Pro Eye. The HTC Vive Pro Eye is a high-end VR headset with integrated Tobii eye-tracking technology, featuring a dual AMOLED 3.5-inch screen, 2,880 × 1,600 pixel resolution, and 120 Hz eye-movement data output frequency, with an accuracy range of 0.5–1.1°, and a tracking field of view of up to 110°, which allows it to accurately capture eye-movement characteristics of the subject. All experimental processes, data processing and analysis are done on a Seven Rainbow X17 Pro Max notebook equipped with an Intel Core i9-13900HX processor and an NVIDIA GeForce RTX 4080 graphics card, ensuring efficient processing of experimental data.

The experimenter was placed in the center of the installation base station space (Figure 2) and a chair was placed to rotate 360° to view the virtual space. A base station was a device that sets the range of virtual space in real space. In this experiment, the base station was mounted face to face on the diagonal ends of the main body for the experimental procedure. The Experiment Manager was placed in close proximity to the subjects and equipment to enable the experiment to be performed. A PC host was used to monitor the status of the experiment.

### 3.5 Data collection and preprocessing

**Figure 3:**
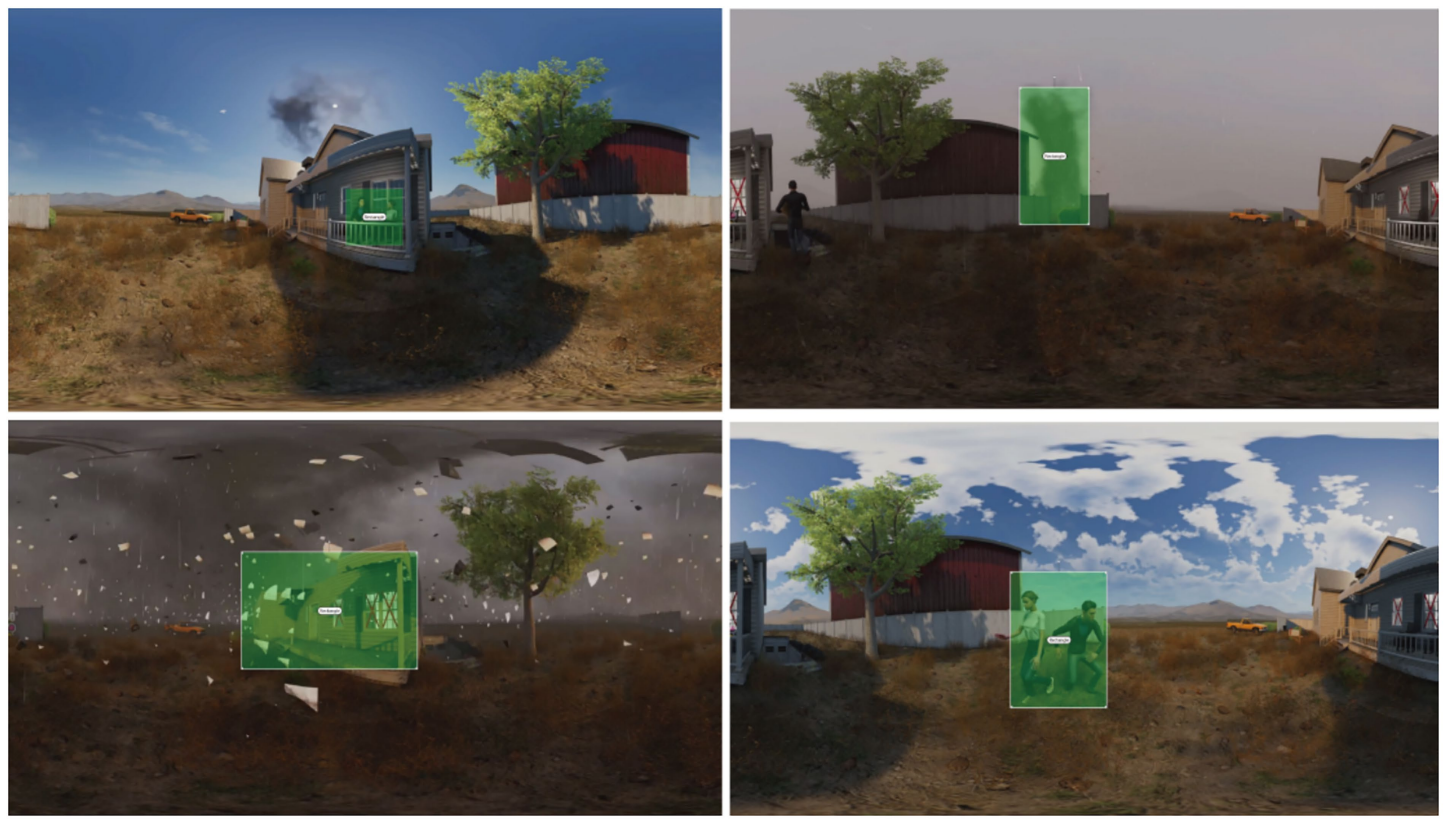
Example of dynamic AOI region partitioning for a movie.

Different eye-tracking metrics can reflect distinct aspects of visual attention and cognitive load. Fixations (Joseph et al., 2021), saccades (Yu et al., 2024), and pupil diameter (Krejtz et al., 2018) are among the most commonly used measures of visual attention and cognitive load. Before outputting the above metrics, the area of interest (AOI) of the film is first delineated. In this project, since the film narrative unfolds in a 360° spatial environment, we delineate the dynamic AOI region (Figure 3) so that it constantly changes according to the development of the film narrative. The scope of the AOI region included characters, a tornado, houses destroyed by the tornado, and other parts that contain information about the main narrative content, eliminating empty scenes where no main narrative content occurs.

Eye-tracking data metrics can help us better understand how viewers interact with movie content. The total gaze duration (TDF) and total sweep duration (TSD) reflect how much attention viewers pay while watching a movie, while gaze count (FC) and sweep count (SAC) provide dynamic information about changes in viewers’ visual attention (Just and Carpenter, 1976). On the other hand, average pupil diameter (APD) and pupil hit rate (PPR) evaluate the effectiveness of the movie in terms of pleasurable experience and emotional response, respectively (Bradley and Lang, 1994). The eye-tracking heat map and the eye-tracking trajectory map enable to visualize the distribution of attention and the visual path of the viewer while watching movies in the VR environment. This visualization tool helps us to identify visual hot-spots in our movies, revealing which scenes or content capture the viewer’s attention the most. At the same time, this visualization tool can reveal how the viewer’s attention shifts in response to movie clips and whether the clips lead to frequent distractions. Therefore, we extracted the total gaze duration, gaze count, total sweep duration sweep count, average pupil diameter, and pupil hit rate for the AOI regions of F1, F2, and F3, respectively. After an initial check of the raw data, we excluded two sets of invalid data (their eye-tracking data was disorganized and illogical), 40 valid datasets were left after excluding invalid data. Data were analyzed using SPSS 29.0.1, including descriptive statistics, ANOVA test and correlation analysis. Finally, the effects of different editing styles on the viewer’s narrative perceptions were explored in conjunction with the generated eye-movement heat maps and eye-tracking trajectory maps.

## 4 Results

### 4.1 Numerical results of eye movement experiments

The results of the one-way ANOVA for TDF, TSD, FC, SAC and APD are shown in Table 2. The analysis of TDF revealed that the F1 group scored significantly higher than both the F2 and F3 groups, with no dramatic difference observed between the F2 and F3 groups [*M*(F1—F2) = 18953.825, *p* = 0.000 < 0.05; *M*(F1—F3) = 18130.975, *p* = 0.000 < 0.05]. Similarly, in the FC results, the F1 group again exhibited hugely higher scores compared to the F2 and F3 groups, while no significant difference was found between F2 and F3 [*M*(F1—F2) = 39.600, *p* = 0.002 < 0.05; *M*(F1—F3) = 45.950, *p* = 0.000 < 0.05]. These findings suggest that the clip-less movie (F1) demonstrates greater visual attractiveness than the edited versions. The TSD analysis mirrored these results, with the F1 group scoring sharply higher than both F2 and F3, and no remarkable difference between F2 and F3 [*M*(F1—F2) = 16632.825, *p* = 0.002 < 0.05; *M*(F1—F3) = 15953.175, *p* = 0.003 < 0.05]. The SAC results displayed a similar trend, with the F1 group scoring enormously higher than F2 and F3, while F2 and F3 showed slight difference [*M*(F1—F2) = 38.275, *p* = 0.000 < 0.05; *M*(F1—F3) = 44.300, *p* = 0.000 < 0.05]. Thus, the unedited movie significantly increased the cognitive load on viewers. For APD, both the F1 and F2 groups scored significantly higher than the F3 group, with no meaningful difference between the F1 and F2 groups [*M*(F1—F3) = 0.3970024, *p* = 0.001 < 0.05; *M*(F2—F3) = 0.5960200, *p* = 0.000 < 0.05]. Likewise, the PPR results indicated that the F1 and F2 groups scored considerably higher than the F3 group, with moderate difference between F1 and F2 [*M*(F1—F3) = 0.3970024, *p* = 0.001 < 0.05; *M*(F2—F3) = 0.5960200, *p* = 0.000 < 0.05]. APD and PPR can reflect the viewer’s pleasure to some extent; the more complex the movie clip, the lower the viewer’s enjoyment. In conclusion, while unedited movie exhibits advantages in visual attractiveness, it also increases cognitive load, and complex editing tends to diminish viewer pleasure.

**Table 2:** One-way ANOVA of eye movement indices in different editing method.

Eye-tracking indicators	Film (I)	Film (J)	Mean difference (I-J)	Std error	Significance	95% CI	
						Lower	Upper
TDF	F1	F2	18953.825*	4244.723	<0.001	8643.86	29263.79
		F3	18130.975*	4244.723	<0.001	7821.01	28440.94
	F2	F1	−18953.825*	4244.723	<0.001	−29263.79	−8643.86
		F3	−822.85	4244.723	1	−11132.81	9487.11
	F3	F1	−18130.975*	4244.723	<0.001	−28440.94	−7821.01
		F2	822.85	4244.723	1	−9487.11	11132.81
FC	F1	F2	39.600*	11.402	0.002	11.91	67.29
		F3	45.950*	11.402	<0.001	18.26	73.64
	F2	F1	−39.600*	11.402	0.002	−67.29	−11.91
		F3	6.35	11.402	1	−21.34	34.04
	F3	F1	−45.950*	11.402	<0.001	−73.64	−18.26
		F2	−6.35	11.402	1	−34.04	21.34
TSD	F1	F2	16632.825*	4742.673	0.002	5113.4	28152.25
		F3	15953.175*	4742.673	0.003	4433.75	27472.6
	F2	F1	−16632.825*	4742.673	0.002	−28152.25	−5113.4
		F3	−679.65	4742.673	1	−12199.07	10839.77
	F3	F1	−15953.175*	4742.673	0.003	−27472.6	−4433.75
		F2	679.65	4742.673	1	−10839.77	12199.07
SAC	F1	F2	38.275*	9.772	<0.001	14.54	62.01
		F3	44.300*	9.772	<0.001	20.56	68.04
	F2	F1	−38.275*	9.772	<0.001	−62.01	−14.54
		F3	6.025	9.772	1	−17.71	29.76
	F3	F1	−44.300*	9.772	<0.001	−68.04	−20.56
		F2	−6.025	9.772	1	−29.76	17.71
APD	F1	F2	−0.01990176	0.01112822	0.221	−0.046544	0.0067405
		F3	0.03970024*	0.01134899	0.001	0.0125294	0.0668711
	F2	F1	0.01990176	0.01112822	0.221	−0.0067405	0.046544
		F3	0.05960200*	0.01247641	<0.001	0.029732	0.089472
	F3	F1	−0.03970024*	0.01134899	0.001	−0.0668711	−0.0125294
		F2	−0.05960200*	0.01247641	<0.001	−0.089472	−0.029732
PPR	F1	F2	−0.04981680*	0.00693032	<0.001	−0.0664088	−0.0332248
		F3	−0.04879688*	0.00706782	<0.001	−0.0657181	−0.0318757
	F2	F1	0.04981680*	0.00693032	<0.001	0.0332248	0.0664088
		F3	0.00101992	0.00777006	1	−0.0175825	0.0196223
	F3	F1	0.04879688*	0.00706782	<0.001	0.0318757	0.0657181
		F2	−0.00101992	0.00777006	1	−0.0196223	0.0175825

***p < 0.001, **p < 0.01, *p < 0.0.

### 4.2 Eye-tracking heat map and eye-tracking trajectory map

**Figure 4:**
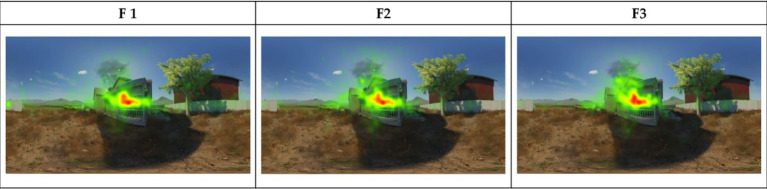
Eye-tracking heat map.

In the eye-tracking heat map, the colored areas represent the extent of the viewer’s attention to the AOI, from green to red, with progressively longer gaze duration. Visual attention, defined as the cognitive process of selectively focusing on specific stimuli while ignoring others (Posner and Cohen, 1980), was measured through gaze duration and fixation density within AOIs. Figure 4 shows that the viewer’s visual attention is most concentrated in F1 and most widespread in F3. In the eye-tracking heat map of F1, areas of visual concentration are prominent, with several hot-spots focused on key narrative parts of the film. These hot-spots show a high density of fixation points, indicating that viewers are paying close attention to key narrative areas when watching F1. Compared to F1, F2 shows a more balanced distribution of the viewer’s gaze and more scattered hot-spots areas. This suggests that there is a more balanced distribution of visual elements or information in F2, with no distinct points to draw the viewer’s attention. In the eye-tracking heat map of F3, the viewer’s attention is more dispersed. This suggests that F3 has relatively little visual attractiveness, resulting in an ineffective focus of the viewer’s attention during viewing.

**Figure 5:**
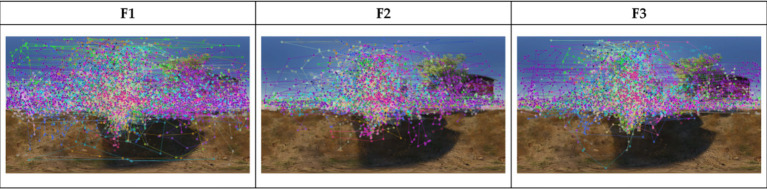
Eye-tracking trajectory map.

Eye-tracking trajectory maps (Figure 5) show the movement of the viewer’s gaze. It can also reflect the temporal sequence of visual stimulus processing in the brain. The trajectory of the viewer’s gaze shows clear and concentrated clusters on the map. Larger clusters tend to point to the main narrative elements, indicating that these areas are effectively capturing the viewer’s attention. In addition, the viewer’s long gaze duration can lead to a high cognitive load. Therefore, larger clusters indicate a longer gaze duration in that area, suggesting a higher cognitive load in those regions. Overall, F1 has the largest area of gaze trajectory clusters, indicating that the viewer’s gaze trajectories are more widespread. This suggests that F1 has the strongest visual attractiveness of the three movies. It also shows that the cognitive load for the viewer is highest in F1. In contrast, F2 and F3 have the smallest area of gaze clusters and more concentrated points of gaze. This suggests that certain narrative areas still attract the attention of the viewer. However, the cognitive load in F2 and F3 may be lower than in F1.

### 4.3 Results of viewer’s emotional experiences questionnaire data

The statistical table of the results of the viewer’s emotional experience questionnaire (Table 3) shows that F1 has the best overall feedback. 67.5% of viewers think F1 has the highest visual fluency, 60% of viewers think F1 has the clearest content. These two indicators show that viewers were provided with a strong sense of plot immersion in F1, and the viewer is relieved of the burden of processing visual information during viewing. In addition, 52.5% of viewers rated F1 as having a high level of visual comfort, 75% of viewers found F1 extremely immersive. This indicates that this film is able to effectively engage the viewers both on an emotional as well as an audio-visual level, so that they understand the storyline in depth. Overall, viewers felt that F1 gave them most compelling emotional engagement of the three films. F2 and F3 are worse than F1 in terms of visual fluency, content clarity, visual comfort, immersion and overall experience. But there is not much difference between F2 and F3. It shows that editing affects the emotional experience of the viewer, regardless of whether or not editing effects are used.

**Table 3:** Statistics of the results of the viewer’s emotional experience questionnaire.

Aspect	Film	Rating 1 count	Rating 1 percentage	Rating 2 count	Rating 2 percentage	Rating 3 count	Rating 3 percentage	Average score
Plot fluency	F1	6	15%	7	17.5%	27	67.5%	2.53
	F2	20	50%	12	30%	8	20%	1.7
	F3	13	32.5%	21	52.5%	6	15%	1.83
Content clarity	F1	8	20%	8	20%	24	60%	2.4
	F2	20	50%	13	32.5%	7	17.5%	1.68
	F3	12	30%	19	47.5%	9	22.5%	1.93
Visual comfort	F1	12	30%	7	17.5%	21	52.5%	2.23
	F2	14	35%	17	42.5%	9	22.5%	1.88
	F3	14	35%	16	40%	10	25%	1.9
Emotional immersion	F1	3	7.5%	7	17.5%	30	75%	2.68
	F2	21	52.5%	14	35%	5	12.5%	1.6
	F3	16	40%	19	47.5%	5	12.5%	1.73
Overall emotional engagement	F1	7	17.5%	10	25%	23	57.5%	2.4
	F2	20	50%	12	30%	8	20%	1.7
	F3	12	30%	19	47.5%	9	22.5%	1.93

## 5 Discussion

This study explores the impact of VR films edited using three specific editing techniques (unedited, hard cuts, and dissolve transitions) on the narrative cognition of VR viewers through three groups of comparative experiments. By combining eye-tracking data with neuroscientific frameworks, particularly regarding the role of the prefrontal cortex and the amygdala, we reveal how these three transition types affect visual attention, emotional experience, and cognitive load. The results support H1: both hard cuts (F2) and dissolve transitions (F3) significantly disrupted viewers’ attention focus, as evidenced by fragmented visual patterns and reduced gaze density in AOIs compared to the unedited condition. By combining eye-tracking data with existing neuroscience theories, this study not only reveals the impact of hard cuts and dissolve transitions on the viewer’s visual attention and emotion, but also provides explanations from a neuroscientific perspective, especially in terms of the role of the prefrontal cortex and the amygdala. The results showed that although edited VR films can be effective in reducing the cognitive load on the viewer, unedited VR film is more effective in capturing the viewer’s attention and enhancing their emotional experience. The continuous stimuli are more likely to keep the viewer focused (Carrasco, 2011). At the neuroscientific level, the amygdala, as a core region for emotional processing, may be continuously activated in unedited VR movies, enhancing emotional reactions (Huang et al., 2023). As a result, in the unedited films, the viewer’s attention can flow more naturally, rather than being forced to refocus because of clip changes (Pecheranskyi, 2023). Compared to traditional films, VR films can enhance the emotional connection between the viewer and the film, increasing the sense of engagement through an embodied experience (Tian et al., 2021). This finding has important theoretical and practical implications for optimizing the narrative structure of VR films and enhancing viewers’ narrative cognition.

To avoid potential order effects that might caused by showing the movies in the same order to all participants, the viewing order of the three movie versions (F1, F2, and F3) was randomized for each participant. Research has shown that the order in which stimuli are presented can influence the experimental results (Sohn et al., 1997). Early contact with certain plot elements may create a priming effect, influencing emotional responses to subsequent content (Henson et al., 2014). This experimental design ensured that the evaluation of each editing style was not influenced by the sequence in which the clips were viewed, thereby enhancing the internal validity of the findings. Our results demonstrate that a completely uncut space–time is more likely to enhance the viewer’s immersion, thus allowing them to focus on the key content. The results of the TDF and TSD data supported these views. When viewers watched the unedited VR films, their attention and emotional responses were significantly more stable. The visual attractiveness of the unedited VR film was further validated by eye-tracking heat maps and eye-tracking trajectory maps. In the images, the viewer’s eyes are focused on key locations in the film’s narrative, emphasizing the focusing effect of the film’s narrative content on the viewer’s visual attractiveness. The tendency of the edited VR film to be more diffuse indicates that the visual attractiveness of the film is weak and does not enhance the viewer’s attention to the content of the film. This counter-intuitive finding suggests that editing, traditionally seen as a tool to guide attention in traditional film, may become a cognitive distractor in immersive VR environments. Unlike flat-screen narratives where visual control is centralized, VR viewers rely more on embodied perception and spatial context. As a result, abrupt scene transitions may disrupt the attentional flow, indicating the need to rethink how traditional editing grammar translates to immersive contexts.

From a psychological perspective, eye movement behavior is a direct external indicator of internal cognitive and emotional states (Rayner, 1998). In heat maps, concentrated fixation points on narrative-relevant areas indicate strong attentional engagement, while smooth and coherent gaze trajectories reflect low cognitive friction, suggesting a fluent narrative processing experience. In contrast, the heat maps for the edited version display more dispersed fixation areas and irregular, erratic eye movement paths, indicating that the editing caused disruptions that required greater cognitive effort to redirect visual attention. These findings correspond to research in the field of neurocinematics, which explores the relationship between cinematic techniques and brain activity (Hasson et al., 2008). In unedited VR films, the continuous visual and narrative flow preserves temporal continuity—a factor that may enhance neural synchrony among viewers and support more coherent processing in brain regions such as the posterior parietal cortex (associated with attention) and the medial prefrontal cortex (linked to narrative understanding and empathy). This neural consistency is reflected behaviorally in more stable eye movement patterns and emotionally in an increased sense of subjective immersion. From a neuroscientific perspective, the prefrontal cortex is crucial in regulating attention and cognitive load. In addition, once visual stimuli enter the brain, they are first processed in the primary visual cortex (V1), which extracts basic features like brightness, edges and orientation. The information is then sent to higher visual areas (such as V4, MT, and IT) for more complex image recognition and spatial construction (Ungerleider and Haxby, 1994). During this process, visual information not only activates the visual cortex but also reaches the amygdala via subcortical pathways, thereby leading emotional responses (Pessoa and Adolphs, 2010). The amygdala is particularly sensitive to emotionally remarkable cues from the visual cortex and is capable of rapidly detecting threatening or affectively charged images, initiating both physiological and emotional reactions (LeDoux, 1996). In VR films, where the immersive visual input is continuous and intense, the connectivity between the visual cortex and the amygdala may become more active, improving the viewers’ emotional engagement and contextual resonance. This interactive mechanism also helps explain why unedited VR films tend to outperform edited versions in visual fluency and emotional immersion. In unedited virtual reality films, the continuous flow of the narrative puts less strain on the dorsolateral prefrontal cortex, allowing for stable attention and fostering a stronger connection to the film. This seamless progression helps the viewer engage with the narrative more easily, free from the distractions of frequent cuts, resulting in a more immersive viewing experience (Hu and Roberts, 2020). “VR in film-making has a stronger effect on human emotional processing during subjective experience and physiological reaction compared with traditional 2D films” (Ding et al., 2018). Viewer questionnaires provided important insights into the emotional value of VR films with different editing styles. Firstly, unedited VR films perform best on metrics such as visual fluency, content clarity and immersion. Compared to edited VR films, the high scores for unedited VR films not only reflect their superior visual presentation, but also mean that viewers are significantly more emotionally invested in their viewing. In emotional processing, the amygdala not only plays a crucial role in regulating emotional responses but also rapidly evaluates the emotional significance of visual stimuli by receiving input from higher visual areas, such as the visual cortex and temporal lobe regions (Morris et al., 1998). Particularly in immersive VR environments, rich complex visual cues activates the amygdala more easily, strengthening its synergy with other brain regions such as the prefrontal cortex and hippocampus, thereby deepening the persistence and intensity of emotional responses. In unedited VR films, the continuous stimuli may lead to more consistent activation of the amygdala, enhancing the viewer’s emotional reactions and engagement with the film. This stronger emotional connection could explain why viewers rated unedited VR films higher for visual fluency, content clarity, and immersion (Mancuso et al., 2023). Viewer’s emotional attention makes them willing to understand the story more deeply (Mouw et al., 2019), and can deepen the memory of a plot (Laird et al., 1982), to facilitate narrative cognition. For the edited VR films (whether using hard cuts or adding transition effects), although they received positive feedback on some aspects, the emotional experience was generally significantly lower than for the unedited VR films, particularly in terms of visual fluency and immersion. Conversely, the emotional experience in edited VR films was generally lower, likely due to the interruption of the immersive experience, which might disrupt the continuity of amygdala activation and weaken the emotional connection to the content. In summary, unedited VR films not only dominate in terms of visual attractiveness, but also significantly enhance the emotional experience of the viewer. Different system qualities determine the emotional nature of the user experience by affecting perceived ease of use and perceived usefulness (Puiu and Udriștioiu, 2024), and given the same systemic quality, this emotional depth is closely related to the viewer’s understanding and identification with the film’s narrative content. This finding suggests that H2 is supported.

However, the unedited VR film (F1) is prone to negative effects on narrative cognition due to cognitive overload (Bradford, 2011). The results of FC, TSD, and SAC values indicate that F1 significantly higher than the edited ones in terms of cognitive load. The high FC values show that viewers frequently move their attention between different visual information. It indicates the complexity of information processing and the use of cognitive resources in this film (Remington and Loft, 2015). F1 also have a sharply higher TSD than the other groups, meaning that the viewer’s eyes stayed on important scenes for longer. This behavior, despite improving the viewer’s understanding of the image, also suggests that they need more cognitive effort to integrate the information. In addition, the SAC values showed that the number of viewer eye jumps was most frequent in F1, as viewers had to quickly switch attention between different visual stimuli to obtain information, thus increasing cognitive load (Posner, 1980). The analysis of the eye-tracking heat maps showed that the visual attention of the unedited VR film was focused on specific regions and that several high-density visual stops are formed in these regions. This suggests that viewers have a higher cognitive load in these regions. Because these areas contain the key plot points or visual elements of the film, it takes longer for the viewer to process and understand this information (Clifton et al., 2016). In the eye-tracking trajectory map, F1’s eye track is much more focused, showing that viewers have a high degree of visual attractiveness to certain scenes. This concentration of vision not only increases the viewer’s engagement, but also increases the cognitive load, as these areas require the viewer to think and understand more deeply (Sweller, 1988). In this process, the sustained activation of the prefrontal cortex aids the viewer in integrating complex information, while the amygdala may serve as a catalyst for emotional reactions (Dixon et al., 2017). In contrast, the oculomotor gaze points and gaze trajectories of the two edited films show a more dispersed pattern, indicating that viewers are less burdened with information processing. In summary, editing will reduce the cognitive load on the viewer. Therefore, H3 has support. However, high cognitive load is not all negative, it has the potential to increase the viewer’s understanding and engagement with the plot. The results of the questionnaire showed that F1 would impose a high cognitive load on the viewers. However, viewers remain highly receptive to the visual fluidity and content clarity of unedited VR films. Therefore, we cannot deny that a high cognitive load will improve viewers’ concentration to some extent, which in turn will enhance their overall viewing experience.

Based on the theory of narrative cognition, this study examines the effects of unedited VR film, hard cut VR film and dissolve edited VR film on viewers’ narrative cognitive abilities from three perspectives: attentional attractiveness, affective experience, and cognitive load. Preliminarily, our eye-tracking results seem to validate the findings of Serrano. Their research is based on cognitive event segmentation theory and explores the possibility of applying film editing techniques from traditional film to VR film. They observed that unnatural editing creates gaps between shots and then interrupts the viewer’s sense of immersion and spatial awareness (Serrano et al., 2017a). This was also found in our study: both hard cuts (F2) and dissolve transitions (F3) distracted viewers from the narrative. Zhang J. et al. (2024) demonstrated that applying the 30° and 180° rules to hard cuts could mitigate spatial disorientation in VR, which aligns with our neuroscientific interpretation: when hard cuts (F2) maintain spatial coherence, the visual cortex processes information more efficiently, reducing cognitive load (Harjunen et al., 2017). For the techniques tested here, we highlight that dissolve transitions (F3) induced fragmented attention due to temporal overlap disrupting spatial continuity, thus requiring avoidance in spatially complex scenes; whereas hard cuts (F2), when adhering to spatial rules, for example 30° principle, reduced cognitive load without severely compromising immersion. Therefore, while both techniques lowered cognitive load relative to unedited VR (F1), their effectiveness depended on spatial management: dissolves demanded stricter constraints than hard cuts. Compared to previous studies, our innovation is to find editing rules that are directly suitable for VR films based on the characteristics of VR films, instead of applying editing techniques from traditional films to VR films. With this in mind, our study highlights the following key points. Firstly, the editing of VR films should focus on the natural transition between shots. Reducing the cognitive load caused by frequent editing helps the prefrontal to process plot information more efficiently, enhancing the viewer’s emotional experience, thus improving their narrative cognition. Therefore, VR film editing should focus on natural transitions within and between scenes. Meanwhile, VR films should try to maintain the integrity of space within the shot and reduce artificial editing, allowing the viewer’s visual cortex to more easily identify the relationship between space and action, thus avoiding cognitive overload. This is a very different approach to traditional films, which use a variety of edits to regulate the pace of the narrative (Carroll et al., 2019) and enhance visual attractiveness (Grushka, 2010). VR films should also try to use smooth, spatially logical scene transitions to avoid distracting the viewer’s attention and interfering with their understanding of the plot. Secondly, creators cannot completely deny the benefits of editing. In VR films, as the 360° space tends to increase the difficulty for viewers to find the main narrative elements and narrative content, proper editing can help the prefrontal cortex allocate attention appropriately, reduce the cognitive load and overcome the challenge (Vallance and Towndrow, 2022). Compared to previous studies, our innovation is to identify technique-specific editing rules by testing hard cuts (F2) and dissolve transitions (F3) in VR, demonstrating that dissolves disrupt spatial continuity through temporal blending, increasing attentional fragmentation, whereas hard cuts maintain coherence when adhering to the 30° rule, enabling efficient visual cortex processing—contrasting traditional films where varied edits regulate narrative pacing (Carroll et al., 2019) and enhance visual attractiveness (Grushka, 2010). For VR, creators should apply spatial rules to hard cuts (F2) for attention guidance (Vallance and Towndrow, 2022), and restrict dissolves (F3) to low-complexity scenes to avoid cognitive interference.

Beyond the narrative and perceptual mechanisms explored in this study, it is also worth briefly considering the potential therapeutic implications of cinematic experiences. Cinematic therapy, a growing field especially in China with academic centers such as Beijing Normal University at the forefront, uses film as a medium for emotional exploration and psychological healing. This perspective builds an interdisciplinary bridge between neuroscience, film editing, and clinical psychology (Mastnak, 2024). Neurocinematics research has demonstrated that specific editing techniques can reliably synchronize viewers’ brain activity. For instance, a EEG study revealed that continuity editing cuts evoke distinct neural responses, indicating synchronized cognitive processing among viewers (Andreu-Sánchez et al., 2021). Such findings suggest that editing techniques not only engage audiences but may also be used to arouse therapeutic or cathartic experiences. The rhythmic and affective patterns induced by cinematic editing may thus be particularly valuable in therapeutic settings, providing a controlled environment for emotional engagement and reflection. Recent research has explored the potential of film editing as a tool for emotional regulation, suggesting its applicability in psychotherapy and education (Grün et al., 2024). While this study did not directly investigate therapeutic outcomes, the underlying mechanisms we explored—such as attentional alignment and emotional resonance—may contribute to future research that integrates neurocinematic tools into cinematic therapy frameworks. A more obvious consideration of these interdisciplinary potentials would be a fruitful direction for further study.

In summary, our comparison of hard cuts and dissolves reveals a neurocognitive trade-off: while both techniques reduce cognitive load, dissolves significantly impair emotional coherence, whereas hard cuts, if spatially optimized, may preserve immersion. For practitioners, this implies that dissolves should be used sparingly in VR, while hard cuts warrant further optimization for spatial continuity.

### 5.1 Limitations and future research

Although our study provides important insights into narrative cognition in VR films, there are still some unresolved limitations that require further critical reflection. First, although we have measured the narrative cognition levels of viewers through visual attention, emotional experience, and cognitive load, we have not yet clearly defined the optimal balance of these factors in narrative perception or clarified the critical thresholds between them. Currently, there is no reasonable formula for assigning weights to these three factors, which reflects a core issue in current narrative cognition research: how to scientifically quantify and balance multiple influencing factors in complex cognitive processes. While VR films, as an art form, rely more on the viewer’s subjective emotions and emotional resonance for understanding and appreciation, we should critically reflect on whether we have overestimated the importance of these subjective factors, neglecting other factors that may influence the viewer’s cognition. In future studies, exploring the dynamic relationships between visual attention, emotional experience, and cognitive load, and developing a more scientifically sound model will be key to addressing this issue.

Furthermore, the logical framework for editing VR films has not yet been fully established. From traditional films to VR films, the mode of editing control is shifting. In traditional films, creators control the viewer’s visual and emotional experience through editing techniques. They use the sequence, rhythm, and duration of shots to guide the viewer’s attention, shape emotional responses, and subtly influence the viewers’ understanding of the narrative. However, in VR films, the viewer’s freedom of movement breaks the editor’s control, meaning that creators need to find new ways to direct the viewer’s attention and emotional flow. This shift itself reveals the challenges in VR filmmaking: How can we engage viewers in the narrative without compromising immersion? This is exactly the direction in which critical research is urgently needed in the current VR film editing theory. Future studies should not just focus on adapting traditional editing techniques, but critically examine the applicability of existing editing techniques in the VR environment and explore entirely new editing methods that better align with the immersive experience of VR.

When collecting the data, we did not consider factors such as the viewer’s subjective preferences and socio-cultural backgrounds. As the current study focuses mainly on exploring the relationship mechanisms between visual attention, emotional experience and cognitive load, and aims to establish a preliminary framework for narrative cognition, this decision was reasonable. However, it also reflects a possible bias in not sufficiently considering the impact of socio-cultural factors and individual differences on the viewer’s reception of VR narratives. Although we made efforts to control variables in the research design and maintained relative consistency in participants’ cultural backgrounds and preferences to minimize the influence of external variables on the analysis of core cognitive processes, we cannot ignore the potential impact of these factors on the viewers’ emotional resonance and narrative understanding. In fact, existing research may underestimate the importance of socio-cultural background in VR narratives. As noted by Serrano et al. (2017a), cultural background can profoundly change a viewer’s response to plot and emotions. Therefore, future research should critically examine the influence of cultural background and individual preferences on VR film narratives and combine cross-cultural comparative studies to explore how these factors modulate narrative perception and understanding at the individual level.

In conclusion, although the current study provides a preliminary framework for narrative cognition in VR films, it also exposes several unresolved issues, particularly regarding editing techniques, cultural background, and individual differences. Future research should not only deepen the understanding of these factors but also critically reflect on how to overcome these issues and further refine the VR film narrative cognition model to better adapt to the ever-changing VR technological environment and viewer demands.

## 6 Conclusion

By analyzing eye-tracking data and questionnaire responses, this study reveals the dual impact of editing techniques on viewers’ narrative cognition in VR films. Unedited VR films promote emotional coherence, driven by sustained amygdala activation, and maintain attention stability, mediated by the prefrontal cortex, significantly enhancing immersion, visual attention, and emotional engagement. However, unedited VR films also induces higher cognitive load. In contrast, edited films effectively reduce cognitive load by guiding attention to key information, facilitating narrative comprehension efficiency. Nevertheless, this comes at the expense of emotional coherence and immersion, particularly evident in fragmented prefrontal cortex activity, reduced amygdala activation, and disrupted neural synchrony for narrative integration. Dissolve transitions were especially detrimental to viewer enjoyment.

From a neurocinematic perspective, these findings underscore the importance of balancing narrative requirements with immersive experience. Creators should adopt a flexible editing strategy that prioritizes seamless transitions to maintain emotional engagement, while strategically using edits to direct attention and reduce cognitive load at critical plot points. Future research should explore the dynamic interplay between PFC and amygdala activation during VR viewing, using advanced neuroimaging techniques, for example FMRI or EEG to further unravel the neural mechanisms underlying narrative comprehension. This approach will not only refine VR film editing practices, but also deepen our understanding of how the brain processes audiovisual narratives in immersive environments.

## Data Availability

The original contributions presented in the study are included in the article/supplementary material, further inquiries can be directed to the corresponding authors.
